# The emergence and transient behaviour of collective motion in active filament systems

**DOI:** 10.1038/s41467-017-00035-3

**Published:** 2017-06-28

**Authors:** Ryo Suzuki, Andreas R. Bausch

**Affiliations:** 1 Lehrstuhl für Biophysik (E27), Technische Universität München, Garching, 85748 Germany; 2 Institute for Integrated Cell-Material Sciences (WPI iCeMS), Kyoto University, Kyoto, 606-8501 Japan

## Abstract

Most living systems, ranging from animal flocks, self-motile microorganisms to the cytoskeleton rely on self-organization processes to perform their own specific function. Despite its importance, the general understanding of how individual active constituents initiate the intriguing pattern formation phenomena on all these different length scales still remains elusive. Here, using a high density actomyosin motility assay system, we show that the observed collective motion arises from a seeding process driven by enhanced acute angle collisions. Once a critical size is reached, the clusters coarsen into high and low density phases each with fixed filament concentrations. The steady state is defined by a balance of collision induced randomization and alignment effects of the filaments by multi-filament collisions within ordered clusters.

## Abstract (web summary)

Self-organization is observed in cytoskeletal systems but emergence of order from disorder is poorly understood. Using a high density actomyosin system, the authors capture the transition from disorder to order, which is driven by enhanced alignment effects caused by increase in multi-filament collisions.

## Introduction

Active systems show a plethora of intriguing spatio-temporal patterns, a remarkable self-organizing process that exhibits structures ranging from cooperative animal group motion^1, 2^ to as diverse as swarming microorganisms^3–5^ or aster-like structures in cytoskeletal systems^6, 7^. To understand the origins of such fascinating structures, theoretical approaches ranging from micro- and mesoscopic studies^8–14^ to hydrodynamic descriptions^15–20^ have been developed. They have been followed by experimental studies in reconstituted^21–25^ and synthetic^26–30^ systems. These approaches focused mainly on the long-time dynamics and the steady state of the pattern formation. More importantly, the lack of information concerning the interaction among constituents have limited our understanding of polar pattern formation processes, especially in biological active systems. For the actomyosin high density motility assay^21, 22, 25^, multi-filament collisions are instrumental for the creation of polar order^31^, yet how the order emerges from a disordered state still remains elusive.

Here, we show that the ordered cluster state in the high density motility assay is characterized by the convergence of filament densities of the low density phase outside the cluster structures to a value five times lower than the critical filament density *ρ*_c_, independent of the starting conditions. For the high density phase, within the clusters, a common density is also found, which is ten time higher than the critical concentration *ρ*_c_. The constant balance between loss and recruitment rates of filaments from and to the cluster phase results in a stable steady state where the total cluster area is preserved. The transition is driven by the emergence of seeds as quantified by the effective binary collision statistics in a system above the critical transition concentration but in a disordered state that is seen well before the time point where polar order structures appear. We identify the self-amplification of the weak effect of the binary collisions at acute angles along with the resulting increased number of collisions at all angles to be the contributing factors that enable the order transition in the active filament system.

## Results

### Formation of seeds and its influence

**Fig. 1:**
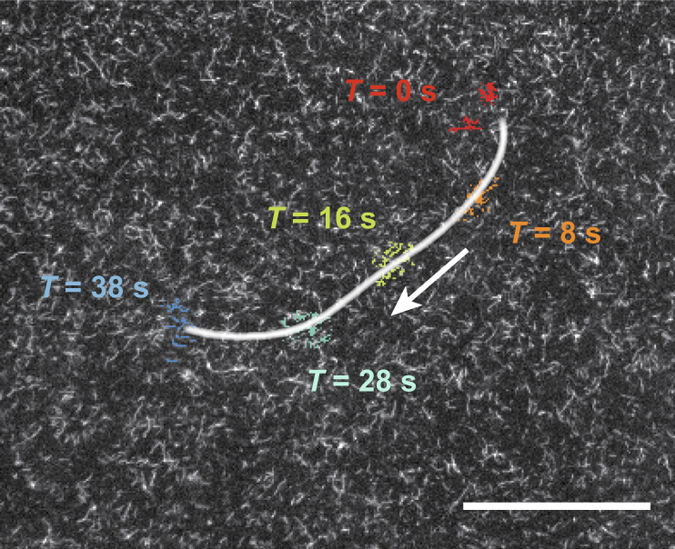
Time trace of ‘seeds’. Premature and unstable small clusters, termed ‘seeds’ contain several filaments, which display a coordinated motion that persists only for a short while. Even before the transition of the system to the distinctive polar ordered state—stable clusters—there are signs of local ordering. However, the direct visualization and hence the quantitative analysis of such seeds are unrealistic due to the absence of significant intensity difference (see Supplementary Movie 1 for the movie of the time trace; the seeds are barely visible). Time trace taken ≈3 min after adding ATP and *ρ* ≈ 5.7 filaments μm^−2^. Scale bar: 50 μm

**Fig. 2:**
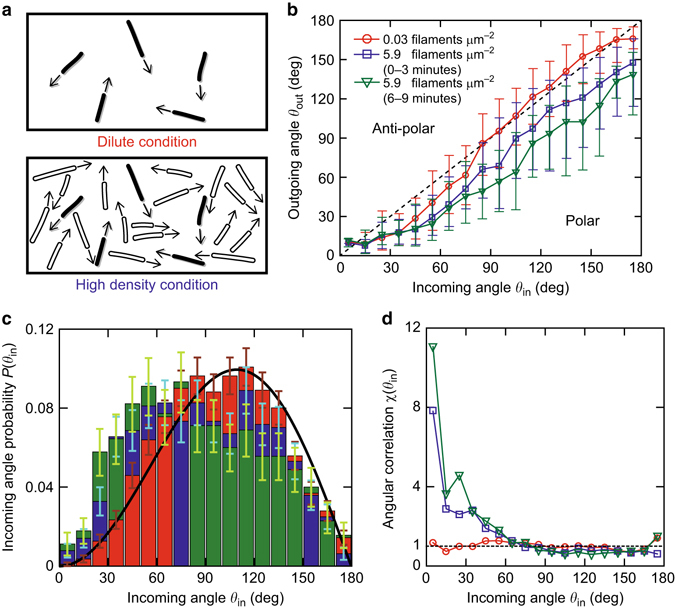
Effective binary collision statistics. **a** Schematic of effective binary collision experiment. While in the dilute condition all filaments are labelled (*top*), at high filament density condition the labelled filaments are put in a pool of unlabelled filaments (*bottom*). The binary collision is evaluated for the labelled filaments. **b** Effective binary collision statistics at high filament density compared to a dilute system (data taken from ref. ^31^). Here, the result for 0.03 filaments μm^−2^ (*red open circles*) and 5.9 filaments μm^−2^ before cluster formation (*blue open squares*: 0–3 min $N_{\rho \, = \, 5.9}^{0 - 3 \, {\rm{min}}} = 641$, *green open downward triangle*: 6–9 min $N_{\rho \, = \, 5.9}^{6 - 9 \, {\rm{min}}}{\rm{ = }}452$) are shown. The *dashed line* represents collisions that are unaffected (*θ*_out_ = *θ*_in_). Above this line (*top-left*), the collisions are anti-polar in nature, whereas below it (*bottom-right*) they are polar. Large enhancement in polar alignment suggests the existence of small patches of locally aligned filaments. This enhancement becomes more apparent later on in time but before the clusters emerge. Error bars: s.d. **c** Incoming angle statistics for the effective binary collision experiment. For higher filament density, the small incoming angles increase. Also with time, small incoming angles increase. *Black solid line* shows the theoretical incoming angle probability, characterized by the Boltzmann scattering cylinder for slender rods (see text). Same colours as in **b**. Error bars: ±(*N*_bin_)^1/2^. **d** Angular correlation *χ*(*θ*_in_) calculated from *P*(*θ*_in_)/*Γ*(*θ*_in_) (see text for detail) using **b** and **c**. The angular correlation emulates a locally aligned patch of filaments as *χ*(*θ*_in_) = 1 + *C*/*θ*_in_, where *C* = 0 (*χ* = 1) corresponds to the molecular chaos assumption. For the dilute case, *C*≈0 (*dashed line* shows *χ* = 1), and a significant correlation for the condition 5.9 filaments μm^−2^, even before emergence of cluster. Same colours and symbols as in **b**

The actomyosin motility assay is comprised of two ingredients: actin filaments and non-processive motor proteins heavy-meromyosin (HMM)^21, 22, 25, 31–33^. By consumption of adenosine triphosphate (ATP), actin filaments move on a lawn of active HMM motors. The collision of filaments results only in a minor change of their direction, as can be seen by the experimentally observed collision tendencies^25, 31^. Increasing the filament density above a critical value *ρ*_c_ results in the emergence of polar clusters^21, 31^. At even higher filament density, density waves appear^21^. The binary interaction between filaments, as quantified by the binary collision statistics in a dilute regime, has been shown to be too weak to enable a transition from a disordered to ordered state. The order transition and the stability of the order relies on multi-filament collisions^31^. To visualize the emergence of the order, we carefully prepare a sample above the critical filament density *ρ*_c_ (=5 filaments μm^−2^), ensuring no pre-alignment of the filaments in the preparation steps. After addition of ATP, very small and highly unstable clusters of aligned filaments can be identified. They consist of about a few tens of filaments and form spontaneously from out of the bulk of disordered filaments and are rapidly annihilated within seconds. We define such unstable structures as ‘seeds’, which are similar to those in classical seeding processes but conceptually differ from spinodal concepts in passive systems since the actomyosin system is active. Such transient small clusters can be observed frequently (Fig. 1), even long before the transition of the system to the distinctive stable-cluster polar ordered state occurs. However, the insufficiency of intensity difference between the seed and bulk, and the seed’s short lived nature does not allow for further quantitative analysis (see Supplementary Movie 1). Yet a quantification of the existence of seeds can be obtained indirectly by determining the effective binary collision statistics in the system before the transition to the ordered phase takes place (see Supplementary Fig. 1 and Supplementary Methods for details on binary collision statistics study). Such information on the effective collision between the constituents provide insights into the transition and emergent patterns^34^. For the condition 5.9 filaments μm^−2^ used here, the time from the addition of ATP to the formation of clusters is of the order $t_{{\rm{order}}}^{\rho = 5.9} \approx 10 - 15{\kern 1pt} {\rm{min}}$. We dilute labelled filaments in a pool of unlabelled filaments (labelled to unlabelled ratio is 1:200). This labelling ratio allows one to determine the effective binary collisions statistics of the incoming angle (*θ*_in_) and resulting outgoing angle (*θ*_out_) between 0–3 and 6–9 min after addition of ATP (Fig. 2a).

Under these conditions, we observe a strong polar alignment tendency in the effective binary collision statistics (Fig. 2b; *blue open square* and *green open downward triangle*) compared to the diluted case (Fig. 2b; *red open circle*) (taken from ref. ^31^). Especially for obtuse incoming angle (*θ*_in_ > 90°), the outgoing tendency is significantly altered to smaller angles. This implies that angular correlations are already present before clusters can be identified, as can be seen directly in the incoming angle statistics *P*(*θ*_in_) (Fig. 2c). At the here studied conditions, the incoming angle probability deviates from the Boltzmann scattering cylinder for slender rods ${\it{\Gamma}} \left( {{\theta _{{\rm{in}}}}} \right) \propto \left| {\sin \left( {{\theta _{{\rm{in}}}}{\rm{/}}2} \right)} \right| \cdot \left| {\sin {\theta _{{\rm{in}}}}} \right|$ ^31, 35, 36^, *black solid line* in Fig. 2c, which is only expected and observed for purely binary collisions of filaments without angular correlations in the system (see Supplementary Methods for more detail).

The degree of deviation from a system absent of angular correlations can be best quantified by a precursor angular correlation function *χ*(*θ*_in_) = 1 + *C*/*θ*_in_ = *P*(*θ*_in_)/*Γ*(*θ*_in_)^31, 37^. Here, *C* is a free parameter that determines the strength of orientational correlations. In the case of *C*= 0, correlations are non-existent, whereas large *C* values correspond to small patches of locally aligned filaments (see Supplementary Methods for detail). Taking *P*(*θ*_in_) (Fig. 2c), which is experimentally obtained, and *Γ*(*θ*_in_) (Fig. 2c; *black solid line*), the higher filament density condition shows clear signs of angular correlations (Fig. 2d); as the system is more polar (higher angular correlations), it is more likely to find collisions that exhibit polar alignment tendencies than collisions in a random system. The change in the collision tendencies and incoming angle statistics over time in the high density pre-cluster regime clearly shows that orientational correlations intensify and alignment among filaments become more and more notable, enabling growth of the polar structures to overcome orientational randomization and hence annihilation of the structures, analogous to seeding processes in equilibrium systems.

### Dynamics of cluster formation

**Fig. 3:**
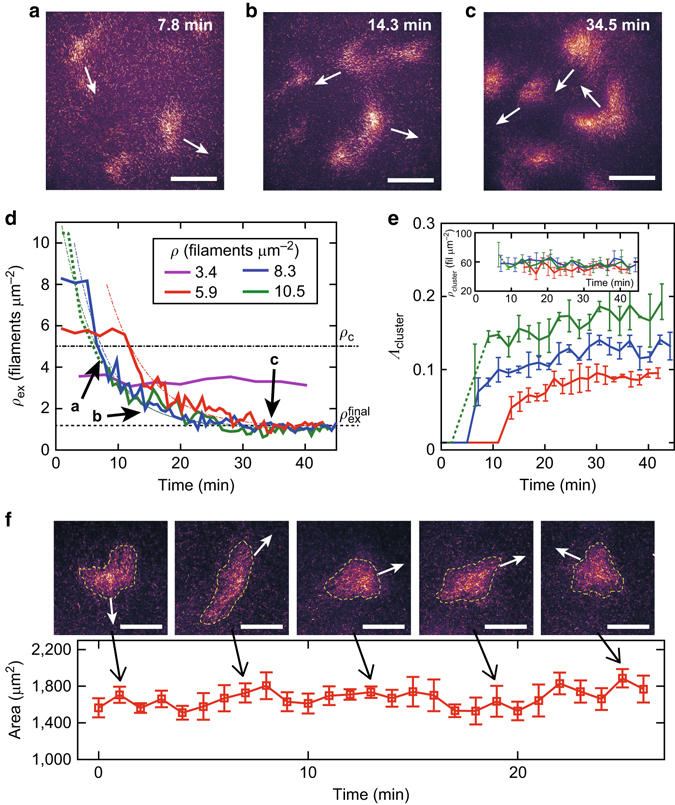
Dynamics of cluster formation. **a**–**c** Time trace of cluster formation for cluster density *ρ* ≈ 8.3 filaments μm^−2^. *White arrows* depict direction of motion for the clusters. **a** 7.8 min, **b** 14.3 min and **c** 34.5 min after initiation of the actin filament motion due to the addition of ATP, as is also indicated in **d**. Scale bars are 100 μm. **d** Dynamics of filament density outside clusters for different initial filament densities. The *dashed line* indicates a final filament density outside the clusters $\rho _{{\rm{ex}}}^{{\rm{final}}}$. The *dash-dot line* is the critical density for order–disorder transition *ρ*_c_. The *dotted line* for *ρ* ≈ 10.5 filaments μm^−2^ indicates a transient density-wave regime. The *dash-dot lines* on top of the data correspond to an exponential fit, where $\rho _{{\rm{ex}}}^{5.9} \propto \exp \left( { - 0.15t} \right){\kern 1pt} {\rm{ + }}{\kern 1pt} 1.12$, $\rho _{{\rm{ex}}}^{8.3} \propto \exp \left( { - 0.19t} \right){\kern 1pt} {\rm{ + }}{\kern 1pt} 1.18$ and $\rho _{{\rm{ex}}}^{10.5} \propto \exp \left( { - 0.17t} \right){\kern 1pt} {\rm{ + }}{\kern 1pt} 1.07$. Here, *t* denotes time. Both the decay tendencies and $\rho _{{\rm{ex}}}^{{\rm{final}}}$ are similar for all conditions. However, the onset times for the emergence of distinctive clusters are dependent on the initial filament density *ρ*. **e** Fraction of cluster occupied area *Λ*_cluster_. Inset: Filament density inside the cluster *ρ*_cluster_, calculated using the experimentally obtained *Λ*_cluster_ and assuming the conservation of total number of filaments. Independent of the initial filament density *ρ*, the clusters have a similar filament density. Same colours as in **d** Error bars: s.d. **f** Area of an individual cluster over time. The constant area over time, along with the constant density of cluster occupied areas demonstrates that with increase in initial filament density *ρ*, the system must increase the number of clusters to sustain the ordered cluster phase. Time 0 corresponds to when the cluster was evidently formed and observable. Filament density condition *ρ* ≈ 5.7 filaments μm^−2^. *Broken yellow lines* show the outline of clusters. Error bars: s.d. within bin, where a bin of 1 min contains 30 images. Scale bars: 50 μm

Prior to the emergence of stable polar structures that persist for considerably longer than the unstable seeds, polar alignment among the filaments develop. Hence, the cluster formation dynamics holds the key to the further understanding on how such coordinated polar ordered structures can emerge. To this end, we investigate how the clusters evolve with time towards the steady state by monitoring the filament density outside the clusters over time (Fig. 3a–c), for different initial filament densities.

We keep the concentration of labelled filaments constant for all conditions, while changing the concentration of unlabelled filaments to vary the total filament density. This ensures a well defined quantitative comparability between the different filament densities. Here, labelled to unlabelled ratio were 1:18, 1:30, 1:42, 1:54, for 3.4, 5.9, 8.3 and 10.5 filaments μm^−2^, respectively. This guarantees a fixed concentration of labelled filaments of 0.18–0.19 filaments μm^−2^ for all conditions. Clusters were identified by treating the raw images with a Gaussian blur filter to smooth the fluorescence signal and then applying a fluorescence intensity cut-off to determine the area and border of the clusters^38^ (see Supplementary Fig. 2 and Supplementary Methods). Applying this method, the density of labelled filaments outside the cluster *ρ*_label_ is determined. This is then rescaled by the labelling ratio *γ*_label_ = (*ρ*_unlabel_ + *ρ*_label_)/*ρ*_label_, where *ρ*_unlabel_ is the density of unlabelled filaments and *ρ*_label_ is the density of labelled filaments which is kept constant. This rescaling enables the comparison between the different filament density conditions, where the filament density outside the cluster is *ρ*_ex_ = *ρ*_label_·*γ*_label_.

The critical filament density *ρ*_c_ for the order–disorder transition is 5 filaments μm^−2^ ^21^, thus the lowest studied filament density condition remains in the isotropic disordered state, while all other conditions develop an ordered cluster phase. For the latter conditions with *ρ* > *ρ*_c_, the total filament density outside the cluster *ρ*_ex_ decays exponentially with a decay time of *τ* = 1/*λ*~5.88 min (decay rate *λ* ~ 0.17(1/min)) to a common value $\rho _{{\rm{ex}}}^{{\rm{final}}}$ (Fig. 3d), where $\rho _{{\rm{ex}}}^{{\rm{final}}}$ is a factor of 5 below the critical concentration *ρ*_c_. In spite of these similarities, the onset of the observable decay of the outside density depends on the starting concentrations and takes longer time for the system with fewer filaments. This delay time to show the first detectable polar ordered structures is ≈13, 6.5 and 4 min for the starting concentrations of 5.9, 8.3 and 10.5 filaments μm^−2^, respectively (Fig. 3d). Before the decay of the outside concentration is observed, the spontaneously formed clusters are unstable and frequently annihilate. Once a few stable clusters of sufficient size have assembled, the system proceeds into the ordered phase with the same time constant for all systems. This observed dependence of the delay time on the filament density is an indication that a nucleation and growth mechanism reminiscent of classical seeding processes is a prerequisite for the order transition to occur.

It is not only the filament density outside the cluster that reaches a common value independent of the initial filament density *ρ*. Using the experimentally obtained fraction of cluster occupied area *Λ*_cluster_ (Fig. 3e) and considering the conservation of total number of filaments in the system, we find that the filament density within the clusters also reach a common value (see Supplementary Methods). The density of *ρ*_cluster_~50–60 filaments μm^−2^, which is a factor of 10 higher than the critical concentration *ρ*_c_ and independent of the initial filament density *ρ* (Fig. 3e, inset). In addition, the area of individual clusters remains constant over time, as observed by tracing the area of a cluster over time for more than 20 min (Fig. 3f). Thus increasing the initial filament density results in an increase of the total area clusters occupy in the system^38^. Since in all studied systems the clusters have the same density of filaments and all the disordered region outside the clusters too have the same filament density, the total area must increase with increasing the starting filament density. The number of clusters and their areas therefore increases with increase in initial filament density condition so that the system can maintain its stability.

### Steady state recruitment capability of clusters

**Fig. 4:**
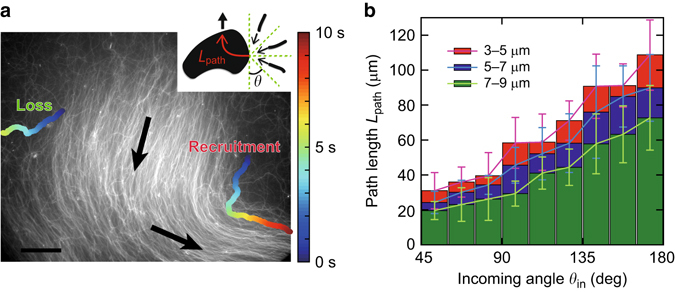
Recruitment capacity of clusters at steady state. **a** Example of recruitment and loss of filaments into and from a cluster. Image shows an overlap of a cluster for 10 s. The direction of cluster motion is depicted by *black arrows*. Scale bar is 30 μm. Inset: Schematic of single filaments entering a polar ordered cluster. The strength of filament recruitment is characterized by the decrease in *L*_path_. The incoming angle of a single filament with respect to the direction of motion of the cluster is *θ*. Here, *θ* = 180° corresponds to a single filament that enters the cluster by colliding head on. **b** Path length *L*_path_ required for a single filament to align to the direction of cluster motion according to its incoming angle *θ*. Long filaments coming into a cluster at an acute angle are persuaded the most. Data taken for events that are persuaded into the general direction of the cluster motion. Error bars: s.d. within each bin. Total number of experiments *N* = 136

We observe that clusters constantly recruit and lose filaments. The stability and sustainability of polar order, depend therefore on a balance between recruitment and loss of the filaments to and from the cluster (Fig. 4a). As individual clusters are able to keep constant areas (Fig. 3f), the recruitment and loss rates are equally balanced. Moreover, the similarities in decay time *τ* of the external filament density *ρ*_ex_ and filament density within the clusters *ρ*_cluster_ for different initial density *ρ* suggests that the combined effect of recruitment and loss of the clusters is universal, irrespective of the starting condition.

The individual strength of a cluster to recruit filaments, can be characterized by observing filaments entering a polar ordered cluster. It can be quantified by the length a single filament travels inside a cluster before it is persuaded into moving in the same direction as the cluster, *L*_path_ (Fig. 4a, inset) (see Supplementary Fig. 3 and Supplementary Methods for details). This path length *L*_path_ increases with increasing incoming angle *θ* (Fig. 4b). Here, only filaments that are convinced by a cluster to go in the same direction were analysed; the majority are recruited. Yet, short filaments coming into the cluster at a very obtuse angle are frequently not persuaded. Also, due to the intrinsic noise of motion of individual filaments also within the clusters, it is not possible to determine the alignment effects for acute incoming angles *θ* ∈ (0°, 45°). Regardless of the incoming angle *θ*, shorter filaments take a longer path to be persuaded to the general direction of the cluster. This is consistent with the fact that shorter filaments, which have a smaller degree of simultaneous collisions at a given filament density (*ρL* ^2^), need a higher density of filaments to create polar order^31^. Short filaments opposing the direction of motion need the longest path of ≈100 μm, during which the direction of the cluster can change.

Hence, the contribution of fast and strong alignment of acute angle collisions seem to dominate the recruitment of bulk filaments into the clusters—defining ‘alignment’ as outgoing angle *θ*_out_ < 30° and using data from Figs. 2b, c, it can be estimated that above *ρ*_c_ before cluster formation, the polar alignment is enhanced by a factor of 3 for early time and a factor of 4 for later time (see Supplementary Fig. 4 and Supplementary Methods for details). The *L*_path_ result suggests that a minimum stable size for clusters is ≈20–40 μm in diameter, where acute angle collisions, which play the key role, can be persuaded. This is a good estimate, as can be seen by comparing with Fig. 3f. A cluster of mean diameter 40–50 μm, which is slightly larger than the aforementioned minimum stable size, is already durable and is stable for a long time.

### Decorrelation of filament trajectories

**Fig. 5:**
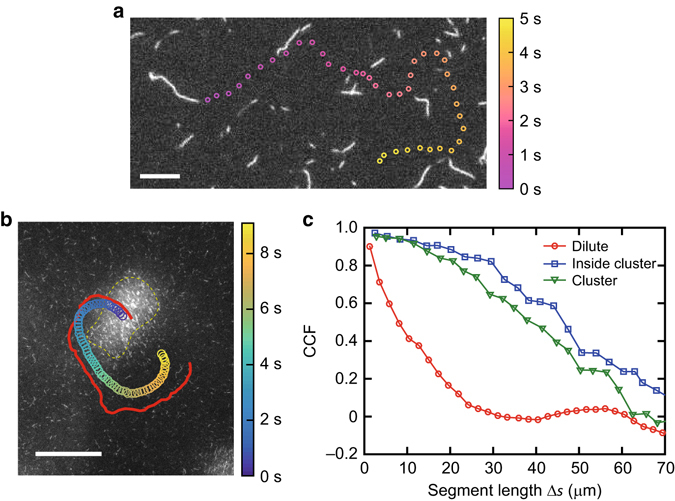
Orientational decorrelation of filament trajectories. **a** Typical trajectory of a single filament in a dilute condition (*ρ* = 0.03 filaments μm^−2^), defined by the head of the filament. Scale bar: 2 μm. **b** Time trace of a filament head trajectory inside a polar ordered cluster (*red solid line*), and the centre of the cluster itself (*open circles*), at filament density *ρ* = 5.9 filaments μm^−2^. The cluster is indicated by a *yellow dashed line*. Scale bar: 50 μm. (**c**) Cosine correlation function (CCF) as a function of segment length Δ*s* (travelled distance) for single filaments in a dilute condition (*ρ* = 0.03 filaments μm^−2^: *red open circles*), inside a cluster (*blue open squares*) and that of clusters themselves (*green open downward triangles*). The persistence of the filament motion inside a cluster is greatly enhanced compared to those in a dilute bath of filaments. Here, *N*_Dilute_ = 38, *N*_Inside_ = 21 and *N*_Cluster_ = 14

The recruitment of filaments and hence the formation of a cluster is always opposed by noise in the system that is induced by the active motion of the motors driving the filaments. Such noise manifest themselves as orientational decorrelation of moving filaments. A single filament in a dilute condition demonstrates a persistent random walk (Fig. 5a), having a characteristic trajectory persistence of approximately a length of the filament itself^31^. On the other hand, the motion of a single filament inside a cluster, and also the cluster itself, persists longer than compared to the dilute case (Fig. 5b). The persistence of a trajectory, $\ell _{\rm{p}}^{{\rm{cluster}}}$ and $\ell _{\rm{p}}^{{\rm{active}}}$, is determined from the cosine correlation function, $\langle \cos \left[ {{\bf{\Theta }}\left( {s{\rm{ + }}\Delta s} \right) - {\bf{\Theta }}(s)} \right] \rangle {\rm{ = }}\exp ( - \Delta s{\rm{/}}2{\ell _{\rm{p}}})$. Here, Θ(*s*) is the tangential angle at the arc length position *s* along the trajectory, Δ*s* is the segment length of the trajectory (travelled distance). This results in a trajectory persistence of a single filament inside a cluster $\ell _{\rm{p}}^{{\rm{cluster}}} \approx$ 27.4 μm, whereas in the dilute case $\ell _{\rm{p}}^{{\rm{active}}} \approx$ 6.0 μm (Fig. 5c). In the dilute case, even if filaments were capable of alignment, the loss of filaments from the aligned structure will be significantly faster than that needed to be recruited (Fig. 4b). Filaments need at least several times their length to align but in the dilute case, their direction will be randomized. On the contrary, above *ρ*_c_, the persistence length is increased to a degree that is comparable to the length needed for persuasion. The clusters are increasing the time in which the filaments can align with the structure. Moreover, the stability of the cluster is possible by staying together longer, where they cannot diffuse orientationally as they are caged by the surrounding filaments and the loss process is diminished. This again corresponds to the importance of multi-filament collisions for the existence of transition to polar ordered clusters.

## Discussion

The observed transition to polar order—from a spatially and orientationally random pool of filaments to the formation of clusters with a common direction of motion and system density inhomogeneity—rely on how a filament can exhibit polar alignment and likewise the capability to maintain such alignment and staying together. Even well before the transition to such polar ordered state, premature and unstable seed like clusters can be seen, which spontaneously emerge and annihilate without reaching any sort of stable structure. Applying an effective binary collision experiment method—dilute labelled filament in a pool of unlabelled filament and comparing the effective binary collision statistics—we find that both the polar alignment and the incoming angle statistics before the formation of clusters differ significantly from those found for the dilute conditions. In addition, the presence of orientational correlations that are absent in the dilute condition also confirms the existence of seeds in the pre-cluster time regime. Though unstable, the seeds are already capable of enhancing the polar alignment by approximately a factor of 4. The binary collision statistics in the dilute case suggests that seeding is achieved mainly by acute angle collisions of *θ*_in_ < 60°, as only at these angles weak alignment effects are observable for binary collisions. At *ρ*_c_, 25% of the whole multi-filament collisions (see Supplementary Fig. 4, $\rho _{{\rm{cluster}}}^{{\rm{early}}}$ condition) are already sufficient for the formation of seeds—which corresponds to 50–60 simultaneous acute angle collisions; *ρ*_c_ *L* ^2^=200–250 ^31^.

Once clusters are formed, the concentration of filaments outside the clusters *ρ*_ex_ decay exponentially to a common value $\rho _{{\rm{ex}}}^{{\rm{final}}}$ that is well below the critical density *ρ*_c_, independent of the initial filament density. The onset of the decay is however dependent on the initial filament density, where fewer filaments take longer to form clusters, and thus demonstrates that the order–disorder transition in the actomyosin assay is nucleation induced—similar to what was suggested by numerical studies^39^. The constant filament density inside clusters over time indicates that the system, regardless of the initial condition above the critical density *ρ*_c_, maintains its stability via increasing the total area of clusters by simply increasing the number of clusters. On a single cluster level, such invariant behaviour is possible as the recruitment and loss of filaments to and from the clusters are balanced. The susceptibility of single filaments to join the cluster is filament length dependent, where longer filaments need a shorter path length *L*_path_ to be persuaded. Since longer filaments simultaneously collide with more filaments than shorter ones, the resulting tendency of decreasing *L*_path_ for longer filaments implies the importance of multi-filament collisions^31^—persuasion is more intense for higher degree of multi-filament collisions. Meanwhile within the cluster, filaments display a highly persistent motion that is a factor of 5 longer compared to that in a dilute condition. Here, the persistence length increases to a value comparable to the length needed for recruitment which also indicates the balance of recruitment and loss, and hence the stability of the cluster structures.

To conclude, once formed and above a critical size, seeds strengthen themselves by their increased efficiency in recruitment of filaments, also of the ones coming in at obtuse angles. The simultaneous minimization of the loss of filaments occurs via the increase of their persistence in motion and the consequent change of incoming collision angle statistics. This is accompanied by the decrease of the filament density outside the cluster $\rho _{{\rm{ex}}}^{{\rm{final}}}$ well below the critical concentration *ρ*_c_ and the emergence of the observed effective collision statistics, which is highly polar in nature. Thus it is the self-amplification of the weak effect of the binary collisions at acute angles which enables the order transition. Also, an increased number of collisions at all angles for higher filament densities creates a synergistic effect which enhances the capability of recruiting filaments and then the pattern formation can proceed, even in the presence of strong active noise produced by the molecular motors. The demixing into a high and low density phase with fixed filament concentrations has the feature of a binodal decomposition, well known for demixing effects driven by energy minimization in passive systems. These findings provide a conceptual basis for further understanding of ordering phenomena in a broader class of active biological systems.

## Methods

### Assay preparation

G-actin solutions were prepared by dissolving lyophilized G-actin obtained from rabbit skeletal muscle^40, 41^ in deionized water and dialyzing against fresh G-buffer (2 mM Tris, 0.2 mM ATP, 0.2 mM CaCl_2_, 0.2 mM DTT and 0.005% NaN_3_) over night at 4 °C. Polymerization of actin was initiated by adding one-tenth of the sample volume of a tenfold concentrated F-buffer (20 mM Tris, 20 mM MgCl_2_, 2 mM DTT and 1 M KCl). HMM were prepared by dialyzing rabbit skeletal muscle against Myosin-buffer (0.6 M NaCl, 10 mM NaH_2_PO_4_, 2 mM DTT, 2 mM MgCl_2_, 0.05% NaN_3_) at 4 °C ^42^. For fluorescence microscopy, fluorescently labelled filaments stabilized with Alexa Fluor 488 phalloidin (Invitrogen) were used.

Flow chambers were prepared by fixing coverslips (Carl Roth, Germany) to microscope slides (Carl Roth, Germany) by parafilm. The coverslips were coated with a 0.1% nitrocellulose solution, which was made by diluting a 2% solution (Electron Microscopy Sciences, Hatfield, PA) in amyl acetate (Roth), and were left to dry over night, prior to constructing the flow chambers. The chamber has a volume of ≈30 μl and its size is typically three orders of magnitude larger than the length of a single filament, to prevent boundary effects. Before an experiment, both actin and HMM were diluted in Assay-buffer (25 mM Imidazolhydrochlorid pH 7.4, 25 mM KCl, 4 mM MgCl_2_, 1 mM EGTA and 1 mM DTT). The flow chamber is incubated with the HMM dilution and then the surfaces are passivated with a BSA solution (10 mg ml^–1^ BSA (Sigma) dissolved in Assay-buffer), prior to the insertion of the actin dilution. To initiate the experiment, 2 mM of ATP dissolved in Assay-buffer is inserted into the flow chamber along with a standard antioxidant buffer supplement GOC (2 mg Glucose-oxidase (Sigma) and 0.5 mg Catalase (Fluka)) to prevent oxidation of the fluorophore. After adding all components, the flow chamber was sealed with vacuum grease (Bayer Silicones).

### Image acquisition

A Leica DMI 6000B inverted microscope was used to acquire data. A 40× (NA: 1.25), 63× (NA: 1.4) and 100× (NA: 1.4) oil objectives were used. Images of resolution 1344 × 1024 pixels were captured with a charge-coupled device camera (C4742-95, Hamamatsu) attached to a × 0.35 or ×1 camera mount.

### Data availability

The data that support the findings of this study are available from the corresponding author upon reasonable request.

## Electronic supplementary material

Supplementary Information

Supplementary Movie 1

Supplementary Movie 2
