# Supplementary material for: The emergence and transient behaviour of collective motion in active filament systems
Source: Nat Commun. 2017 Jun 28;8:41. doi: 10.1038/s41467-017-00035-3 (PMC5489493; doi:10.1038/s41467-017-00035-3)
Supplement: Supplementary file 1 — Supplementary Information [file 41467_2017_35_MOESM1_ESM.pdf]

File Name: Supplementary Information

Description: Supplementary Figures, Supplementary Methods, and Supplementary References

File Name: Supplementary Movie 1

Description: Footage of time trace of ‘seeds’

File Name: Supplementary Movie 2

Description: Footage of recruitment and loss

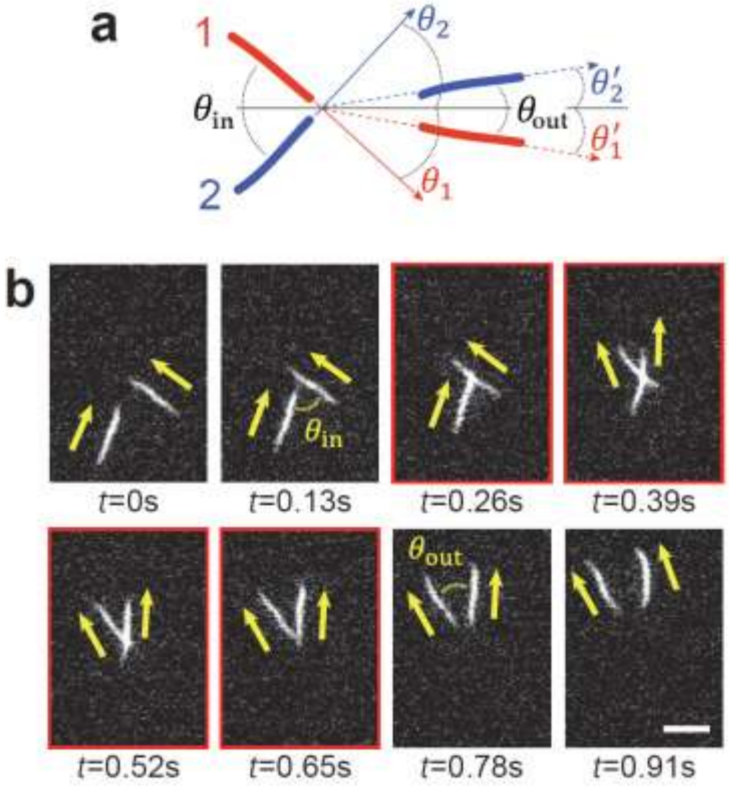

Supplementary Figure 1. (a) Definition of scattering geometry. (b) Example of a collision event. The images inside the red boxes correspond to those during a collision event. The incoming angle θ_{in} is obtained from the image 1 frame before the two filaments touch each other. Similarly, the outgoing angle θ_{out} is taken from the image where the two filaments have parted each other. Scale bar is 2μm and applies to all images.

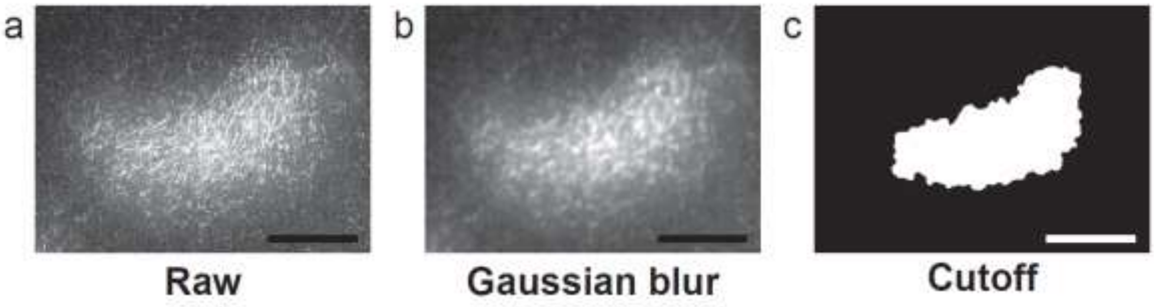

Supplementary Figure 2. Cluster identification. (a) An example of a raw image of a cluster. (b) Raw image treated with Gaussian blur (radius: 2) to make the fluorescence intensity smooth. (c) Based on a fluorescent intensity cut-off, a binary image is obtained from the Gaussian blurred image so as to identify the border and hence the area of the cluster. For all clusters, the same cut-off was applied. Scale bars: 50μm.

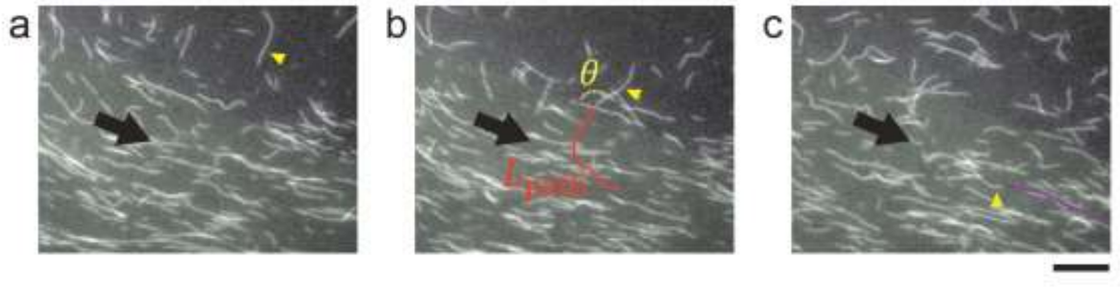

Supplementary Figure 3. Example of filament recruitment of a cluster. (a) A filament, which is indicated by the yellow triangle, is about to enter a cluster region (bottom green part) where the flow of the filaments are from left to right, depicted by the black arrow. (b) Once the filament reaches the cluster boundary, the incoming angle θ is obtained with respect to the filament that is collided by the incoming single filament. Path length L_{path} is determined as the length until the incoming filament aligns with the surrounding filaments. The trajectory for which L_{path} is measured is obtain via hand by tracking the filament head. (c) Trajectory of the filament after L_{path} is obtained. It shows a persistent motion in the same direction as the flow. Scale bar is $10\mu\mathrm{m}$ and applies to all figures (a)-(c).

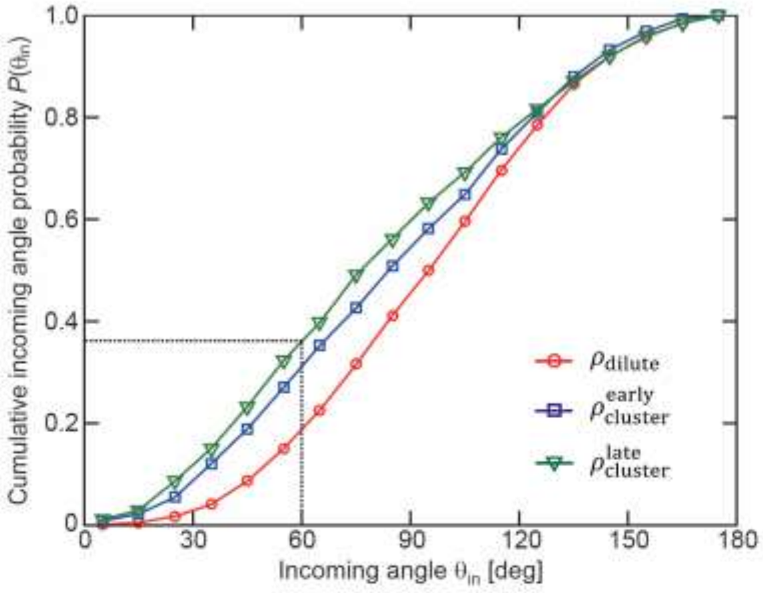

Supplementary Figure 4. Cumulative plot of Fig. 2c. Black broken lines show the example for $\rho_{\mathrm{cluster}}^{\mathrm{late}}$, where alignment (in this condition $\theta_{\mathrm{in}} < 60°$) can be seen for approximately 36% of the total collision events.

Supplementary Methods

Binary collision statistics

A. Obtaining binary collision statistics from experiment

To determine the incoming angle (θ_{in}) and outgoing angle (θ_{out}) for the collision events, the microscopy images were first transformed into binary images. From these binary images, the filaments were identified by skeletonization, using Matlab with a standard library "bwmorph". Then, the coordinates of the filament contour were extracted by use of a cubic spline fit. The obtained coordinates were used to determine θ_1, θ_2 and θ_1', θ_2', hence $\theta_{\mathrm{in}} = \theta_1 - \theta_2$, $\theta_{\mathrm{out}} = \theta_1' - \theta_2'$ [Supplementary Fig. 1a].

A collision is detected when the images of two filaments intersect [Supplementary Fig. 1b]. The incoming angle θ_{in} is obtained from θ_1 and θ_2, 1 frame (0.13sec) before the detected collision event. The end of a collision is detected when the filaments cease to intersect. Here, only binary collisions are studied and all collisions involving more than 3 filaments were discarded.

B. Boltzmann scattering cylinder for slender rods

A Boltzmann scattering cylinder for rods $\Gamma(\theta_{\mathrm{in}})$ describes the frequency in which collisions between propelled rods of constant speed v_0 occur with respect to the relative angle θ_{in} and can be derived from geometric considerations [1, 2]. In the experiments, constant speed of the filament motion is ensured by applying high ATP concentrations. The Boltzmann scattering cylinder for rods $\Gamma(\theta_{\mathrm{in}})$ as a function of the incoming angle θ_{in} is given by

$$\Gamma(\theta_{\mathrm{in}}) = 4dv_0 \left|\sin\left(\frac{\theta_{\mathrm{in}}}{2}\right)\right| \left(1 + \frac{\xi-1}{2}|\sin\theta_{\mathrm{in}}|\right), \quad \text{(S1)}$$

where L is the rod length, d is the rod diameter and $\xi = L/d$ denotes the aspect ratio of the filament. For actin filaments in the experiments, $\xi \sim 1000$, so we can apply the slender rod assumption, $L \gg d$. Thus for slender rods, Eq. S1 becomes

$$\Gamma(\theta_{\mathrm{in}}) = 2Lv_0 \left|\sin\left(\frac{\theta_{\mathrm{in}}}{2}\right)\right| \cdot |\sin\theta_{\mathrm{in}}|. \quad \text{(S2)}$$

The incoming angle statistics $P(\theta_{\mathrm{in}})$ is compared to the functional form of the Boltzmann scattering cylinder for slender rods (see main text Fig. 2c) which, as can be inferred by Eq. S2, depends solely on the incoming angle θ_{in}. Filament properties such as L, d and v_0 only sets the magnitude of Γ.

C. Angular correlation

To imitate the existence of locally aligned filaments or seeds in the framework of binary collisions, angular correlations are implemented. Such angular correlations can be emulated by the function $\chi(\theta_{\mathrm{in}}) = 1 + C/\theta_{\mathrm{in}}$, where C is a free parameter that determines the strength of orientational correlations [3, 4]. It is a measure of how strong the incoming angle probability $P(\theta_{\mathrm{in}})$ deviates from the Boltzmann scattering cylinder $\Gamma(\theta_{\mathrm{in}})$ that represents no angular correlations. Hence, $\chi(\theta_{\mathrm{in}}) = P(\theta_{\mathrm{in}})/\Gamma(\theta_{\mathrm{in}})$. For

$C = 0$, $P(\theta_{\mathrm{in}}) = \Gamma(\theta_{\mathrm{in}})$, so correlations are absent and resembles molecular chaos. Whereas, large C-values correspond to small, locally aligned filament seeds. Here, smaller incoming angle θ_{in} increases its probability, hence more polar alignment.

Using the experimentally obtained $P(\theta_{\mathrm{in}})$ (see Fig. 2c in main text) and Eq. S2 (black solid line in Fig. 2c), $\chi(\theta_{\mathrm{in}})$ is derived. For the dilute case, $\chi \approx 1$ for all incoming angles θ_{in}, which results in $C \sim 0$ and hence no angular correlations (molecular chaos). For the high filament density case, a large C-value can be seen (increase in χ for small incoming angles), indicating the birth of angular correlations within the system.

Dynamics of cluster formation

A. Identification of clusters

To identify clusters, raw images [Supplementary Fig. 2a] were first treated with a Gaussian blur filter (radius: 2 pixels) [Supplementary Fig. 2b], following the procedure in Ref. [5]. The filtering process made the fluorescence signal profile smooth, making the identification of the cluster border simpler when applying an intensity cut-off to obtain a binary image of clusters [Supplementary Fig. 2c]. Here, a same cut-off value was used for all images.

B. Filament density within clusters

To determine the filament density within clusters [Fig. 3(e), inset], the conservation of total number of filaments was assumed. This is only valid in the first hour after the initiation of actin filament motion by adding ATP since after this period, although slowly, actin filaments start to dissociate from the HMM due to depletion of ATP. The above assumption gives $\rho = \rho_{\mathrm{cluster}}\Lambda_{\mathrm{cluster}} + \rho_{\mathrm{ex}}(1 - \Lambda_{\mathrm{cluster}})$.

Filament recruitment and path length

A. Determining L_{path}

Incoming angle θ is obtained with respect to the filament that is collided by the incoming single filament and path length L_{path} is determined as the length until the incoming filament aligns with the surrounding filaments [Supplementary Fig. 3]. The trajectory for which L_{path} is measured, is obtain via hand by tracking the filament head. Filaments that were not persuaded into the clusters were discarded from the results, though such event is not common.

B. Calculating alignment enhancement

In this section, for the sake of convenience, we write the filament densities used in Fig. 2 as ρ_{dilute} for the dilute case, $\rho_{\mathrm{cluster}}^{\mathrm{early}}$ for the pre-cluster high density case between 0-3 minutes and $\rho_{\mathrm{cluster}}^{\mathrm{late}}$ between 6-9 minutes.

Defining alignment as outgoing angle $\theta_{\mathrm{out}} < 30°$, from Fig. 2b the necessary incoming angle range to achieve this is approximately $\theta_{\mathrm{in}} < 45°$, $< 55°$, and $< 60°$ for ρ_{dilute}, $\rho_{\mathrm{cluster}}^{\mathrm{early}}$ and $\rho_{\mathrm{cluster}}^{\mathrm{late}}$,

respectively. Associating these values to Fig. 2c, approximately 8%, 27%, and 36% of the total collisions allow for alignment for ρ_{dilute}, $\rho_{\mathrm{cluster}}^{\mathrm{early}}$ and $\rho_{\mathrm{cluster}}^{\mathrm{late}}$, respectively. Supplementary Fig. 4 shows Fig. 2c as a cumulative probability. Take $\rho_{\mathrm{cluster}}^{\mathrm{late}}$ as an example. Collision events with $\theta_{\mathrm{in}} < 60°$ correspond to 36% of the whole collision events, as is depicted by the black broken lines in Supplementary Fig. 4. Hence, the alignment is enhanced by a factor of approximately 3 and 4 for $\rho_{\mathrm{cluster}}^{\mathrm{early}}$ and $\rho_{\mathrm{cluster}}^{\mathrm{late}}$, respectively.

Supplementary References

[1] Weber, C. A., Thüroff, F. & Frey, E. Role of particle conservation in self-propelled particle systems. *New J. Phys.* **15**, 045014 (2013).

[2] Thüroff, F., Weber, C. A. & Frey, E. Critical assessment of the Boltzmann approach to active systems. *Phys. Rev. Lett.* **111**, 190601 (2013).

[3] Hanke, T., Weber, C. A. & Frey, E. Understanding collective dynamics of soft active colloids by binary scattering. *Phys. Rev. E* **88**, 052309 (2013).

[4] Suzuki, R., Weber, C. A., Frey, E. & Bausch, A. R. Polar pattern formation in driven filament systems require non-binary particle collisions. *Nature Phys.* **11**, 839-844 (2015).

[5] Schaller, V. & Bausch, A. R. Topological defects and density fluctuations in collective moving systems. *Proc. Natl. Acad. Sci.* **110**, 4488-4493 (2013).
